# Accuracy of the doses computed by the Eclipse treatment planning system near and inside metal elements

**DOI:** 10.1038/s41598-022-10072-8

**Published:** 2022-04-08

**Authors:** Bartosz Pawałowski, Adam Ryczkowski, Rafał Panek, Urszula Sobocka-Kurdyk, Kinga Graczyk, Tomasz Piotrowski

**Affiliations:** 1 Department of Medical Physics, Greater Poland Cancer Centre, Garbary 15, 61-866 Poznan, Poland; 2 Department of Technical Physics, Poznan University of Technology, Poznan, Poland; 3 Department of Electroradiology, Poznan University of Medical Sciences, Poznan, Poland; 4 Medical Physics and Clinical Engineering, Nottingham University Hospitals NHS Trust, Nottingham, UK; 5 School of Medicine, University of Nottingham, Nottingham, UK; 6 Faculty of Health Sciences, Calisia University, Kalisz, Poland

**Keywords:** Implants, Applied physics, Imaging techniques, Cancer, Computed tomography

## Abstract

Metal artefacts degrade clinical image quality which decreases the confidence of using computed tomography (CT) for the delineation of key structures for treatment planning and leads to dose errors in affected areas. In this work, we investigated accuracy of doses computed by the Eclipse treatment planning system near and inside metallic elements for two different computation algorithms. An impact of CT metal artefact reduction methods on the resulting calculated doses has also been assessed. A water phantom including Gafchromic film and metal inserts was irradiated (max dose 5 Gy) using a 6 MV photon beam. Three materials were tested: titanium, alloy 600, and tungsten. The phantom CT images were obtained with the pseudo-monoenergetic reconstruction (PMR) and the iterative metal artefact reduction (iMAR). Image sets were used for dose calculation using an Eclipse treatment planning station (TPS). Monte Carlo (MC) simulations were used to predict the true dose distribution in the phantom allowing for comparison with doses measured by film and calculated by TPS. Measured and simulated percentage depth doses (PDDs) were not statistically different (*p* > 0.618). Regional differences were observed at edges of metallic objects (max 8% difference). However, PDDs simulated with and without film were statistically different (*p* < 0.002). PDDs calculated by the Acuros XB algorithm based on the dose-to-medium approach best matched the MC reference regardless of the CT reconstruction methods and inserts used (*p* > 0.078). PDDs obtained using other algorithms significantly differ from the MC values (*p* < 0.011). The Acuros XB algorithm with a dose-to-medium approach provides reliable dose calculation in all metal regions when using the Varian system. The inability of the AAA algorithm to model backscatter dose significantly limits its clinical application in the presence of metal. No significant impact on the dose calculation was found for a range of metal artefact reduction strategies.

## Introduction

An increasing number of patients with metallic implants are treated with radiotherapy and so it is important to better understand the impact of these elements on the treatment process. The proximity of a metal object causes streaking image artefacts observed in the computed tomography (CT) scans deteriorating diagnostic quality and ability to confidently delineate structures such as organs at risk and tumours^1–3^. Misrepresentation of CT numbers can also cause errors in calculated linear attenuation coefficients leading to significant errors in dose calculation in affected areas^4^. The simple density override method, covering the affected area by contour with manually corrected CT numbers, can reduce the dose calculation error^5^. However, this method doesn’t improve visualization of areas affected by artefacts, which is important for accurate delineation of anatomical structures. Several metal artefact reduction methods were proposed, such as the iterative metal artefact reduction (iMAR) algorithm^6–8^, the dual-energy method (enabling the pseudo-monoenergetic reconstruction (PMR) of CT images created for specified photon energy)^9^, and a unique technique based on the use of megavoltage CT imaging on the tomotherapy units^10,11^. Combinations of PMR and iMAR methods have been previously investigated and demonstrates significant reduction of metal artefacts and low CT number errors observed in the vicinity of dense materials^12^. Besides errors due to the presence of artefacts, there is also doubt related to the precision of dose calculation in the presence of high-density materials^13^. These metal elements lead to higher beam attenuations and reveal interface phenomena caused by backscatter radiation^14^. For precise dose calculation, these perturbations have to be accounted for. Currently, only algorithms utilising the medium for dose transport and calculation can model this accurately^15^. However, there is still a lack of comprehensive analysis of different metal artefact reduction methods and their impact on algorithms used for dose calculations. Many authors verified the impact of metals on the radiotherapy process, however, most of them used only one metal which can be insufficient for understanding the behaviour of the algorithms and their limitations^16–18^. Our work focused on obtaining a Monte Carlo validated simulation of three metals with different, clinically relevant densities and comparing them with different dose algorithm calculations using a range of MAR methods. In particular, we assess: (1) the accuracy of dose computation using Acuros XB and AAA (analytical anisotropic algorithm) calculation algorithms near and inside the metal structures, and (2) the impact of different methods for metal artefact reduction in CT on the accuracy of doses computed through these algorithms.

## Materials and methods

### Dose measurements

**Figure 1:**
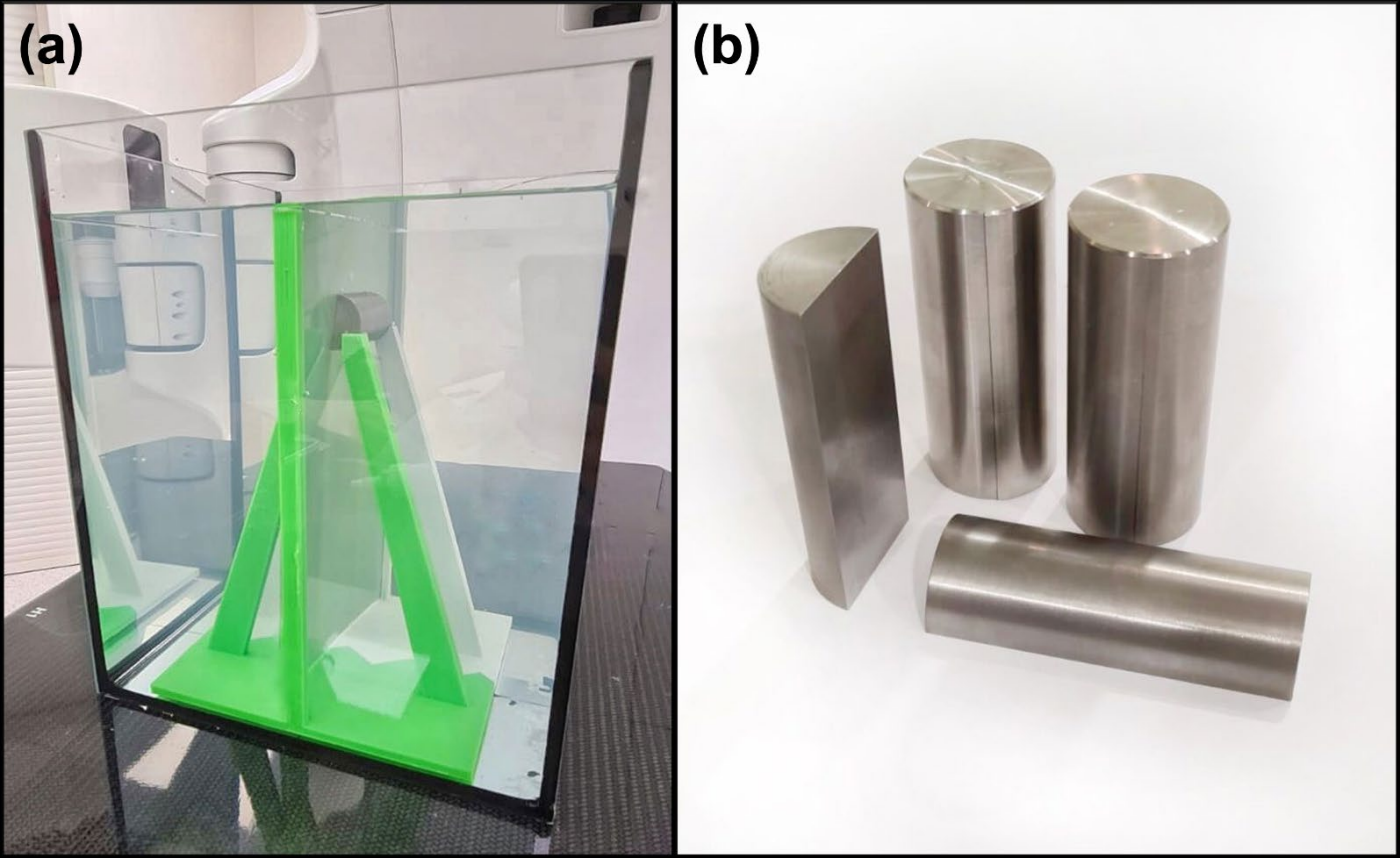
(**a**) A photograph of the water phantom with submerged holder made in 3D printing technology for a Gafchromic film and metallic inserts. (**b**) A cut in a half metal insert made of titanium, alloy 600 and tungsten used in this study.

A 25 × 25 × 30 cm^3^ water phantom with holder for Gafchromic film and metal inserts was fabricated (Fig. 1a). A 3D printed holder was designed to allow for the placement of the Gafchromic film parallel to the beam axis and to fix the removable cylindrical inserts at a precisely defined depth. The distance between the top edge of the Gafchromic film and the center of the insert was 5 cm.

Specific information about phantom components:

1. Phantom holder: Original Prusa i3 MK3S + 3D printer (Prusa Research; Prague, Czech Republic) was used to print the phantom holder in fused deposition modeling technology with polylactic acid filament^19,20^.
2. Inserts: The cylindrical inserts (2.8 cm diameter and 7 cm length), consisting of two halves (Fig. 1b), were made from three high-density materials: titanium, alloy 600, and tungsten with physical densities of 4.5, 8.5, and 19.4 g/cm^3^, respectively^12^. The dimensions of inserts allowed to position them in both dosimetric and CT calibration (Virtual Water™, described below) phantoms.
3. Film: Self-evoking EBT3 Gafchromic films (Ashland Inc., Wilmington, Delaware, USA). The films are made of an active layer with a thickness of 28 μm, located between two layers of matte-polyester substrates with a thickness of 125 μm. These films allow to measure the doses (optimal range from 0.2 to 10 Gy) obtained by radiation beams with energies ranging from 100 to 18 MeV. The film`s response is independent of temperature, atmospheric pressure, and the direction of the irradiation beam^21,22^. To avoid delamination and the water immersion effect, whole EBT3 sheets (20.3 cm × 25.4 cm) were placed in the 3D printed watertight holder.

The phantom including EBT3 film insert was filled with water up to the top edge of the film (Fig. 1a) and positioned on the accelerator couch. The source to phantom water surface distance, SPD, was 100 cm for all measurements. Central-axis percentage depth doses (PDD) were measured and simulated for a 6 MV photon beam produced by the TrueBeam accelerator (Varian Medical Systems, Palo Alto, USA). Films were irradiated with a dose of 5 Gy defined at the point of maximum dose located at 15 mm below water surface. The beam field size was 10 cm × 10 cm oriented perpendicular to the water surface and the central axis (CAX) of the beam was in the middle of the film.

For the dose calibration curve, EBT3 film was cut into nine pieces (3 cm × 3 cm) and irradiated with a photon beam (10 cm × 10 cm, 6 MV) in ranges from 1 to 10 Gy.

The films were scanned 30 h after being irradiated using the Epson Perfection 750 Pro scanner (Seiko Epson Corporation, Japan) with the following parameters: no color correction, transmission mode, portrait orientation, 48-bit Red–Green–Blue (RGB)^23^. The scan resolution was 72 dpi for calibration and measurements. Scans were then analysed using Film Analyze 1.8 (PTW Freiburg, Freiburg, Germany; single red channel analysis from RGB).

### Dose calculations

**Figure 2:**
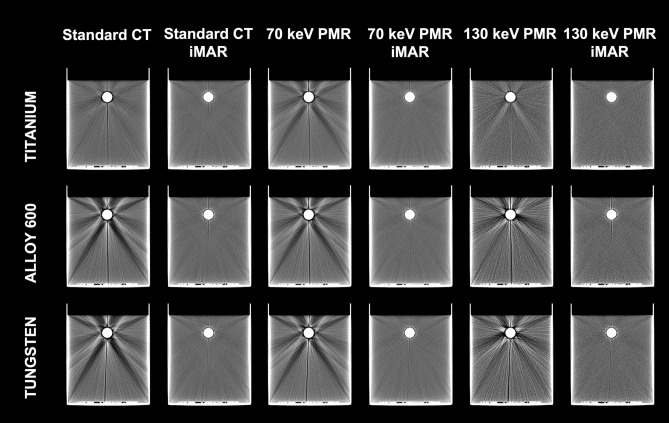
CT images of the phantom with titanium, alloy 600, and tungsten inserts obtained using a range of reconstruction modes. *PMR *pseudo-monoenergetic reconstruction, *iMAR *iterative metal artefact reconstruction.

The phantom images were acquired using a Somatom Definition AS scanner (Siemens Medical Solution, Erlangen, Germany). Two sets of CT images were obtained: (1) standard CT pelvis protocol (120 kV, 270 mAs, 0.6 pitch, 64 × 0.6 mm acquisition, 3 mm slice thickness, 2.0 mm increment, kernel B30s, extended CT scale) and (2) a dual-energy mode based on the two consecutive scan technique (first/second scan: 80 kV, 540 mAs/140 kV, 128 mAs; 0.6 pitch, 64 × 0.6 mm acquisition, 3 mm slice thickness, 2.0 mm increment, kernel B30f., extended CT scale) and used to obtain 70 and 130 keV PMR sets. The standard CT and 70 and 130 keV PMRs were reconstructed twice for each insert: once with and once without the iMAR algorithm (Siemens Medical Solution, Erlangen, Germany) (Fig. 2).

Eclipse v.16.0 (Varian Medical Systems, Palo Alto, USA) treatment planning system (TPS) was used in the study. The doses for each plan (each CT reconstruction) were normalised to the maximum depth and calculated by two available options: Acuros XB v.16.1.0 and AAA (i.e., analytical anisotropic algorithm) v.16.1.0 algorithms. In the case of Acuros XB calculations, two approaches: dose-to-medium and dose-to-water were considered. AAA calculations were based on CT density calculated by CT scanner and real CT value estimated from density. The spatial resolution of 1 mm was used for all calculations.

Six energy-dependent conversion curves (i.e., for each energy of CT reconstruction and for each class of calculation algorithm) were prepared for dose calculations. The curves were obtained using Virtual Water™ phantom (Gammex RMI, Middleton, WI, USA), with various inserts and different tissue densities^12^. The Virtual Water™ phantom was scanned using the same parameters as for the water phantom. Images were reconstructed for standard CT, 70 keV PMR, and 130 keV PMR series.

The segment high-density material option, available in the Eclipse TPS, was used for the insert contouring purposes. This tool finds and outlines structures with mass densities larger than 3 g/cm^3^. For the AAA algorithm, three energy-dependent CT numbers to electron density conversion curves relative to water were obtained. The whole range of CT numbers reconstructed in the insert during imaging was used for dose calculation by the AAA approach based on CT density. The real CT approach, used for dose calculation, manually overrides the value of the CT number with the mean value from the whole range of CT numbers detected in the insert. Conversion curves were also calculated for Acuros XB. The Acuros XB-13.5 material table was used for the Acuros material assignment including titanium alloy (titanium insert), stainless steel (alloy 600), and gold (tungsten). Tungsten material is not currently available for Acuros XB, and gold was used instead due to its comparable density (19.3 g/cm^3^ and 19.4 g/cm^3^ for gold and tungsten respectively).

### Monte Carlo simulations

The Geant4 toolkit v.10.05.p01 was used for the Monte Carlo (MC) simulation of the dose distribution in our phantom for each insert (parallel computations, 5 dual Xeon processors, 32 GB RAM each). As a primary generator, data from phase space files provided by Varian were used^24^. Fifty-five files for the 6 MV photon beam were used in total, each one was iterated twenty times. Two phantom configurations were modelled for dose calculations: (1) MC_mea_ with the information of dose deposited in the film corresponding to the measurement condition in the water-filled phantom with the EBT3 film placed between the two halves of the insert, and (2) MC_pla_ corresponding to dose calculation in Eclipse TPS without the film present. For MC_pla_, the information of dose deposition, unlike MC_mea_, was not collected for the film but in water and the insert. Simulations in both configurations were performed under the same conditions matching experimental irradiation conditions described earlier.

### Data analysis

The PDDs obtained from direct measurements, MC simulations, and Eclipse TPS calculations were compared on depths ranging from 15 to 85 mm, where 0 mm corresponds to the water surface. In particular, the PDDs comparisons were made between: (1) experimental, measured (EBT3) versus simulated MC_mea_ (validation of MC simulated PDD), (2) MC_mea_ versus MC_pla_ (influence of film on PDD) and (3) MC_pla_ versus Eclipse TPS (comparison of MC simulated and TPS calculated PDDs for different algorithms and for different reconstruction methods).

The comparison was made in five regions:

1. water in front of the insert (15–34 mm),
2. input edge of the insert ± 2 mm (34–38 mm),
3. the insert (38–62 mm),
4. output edge of the insert ± 2 mm (62–66 mm),
5. water behind the insert (66–85 mm).

Mean dose differences were calculated for all regions and PDDs. Kolmogorov–Smirnov and Pearson tests were used to test differences between means and correlations with a 0.05 significance level.

## Results

### EBT3 film measurements versus MC_mea_ comparison

**Figure 3:**
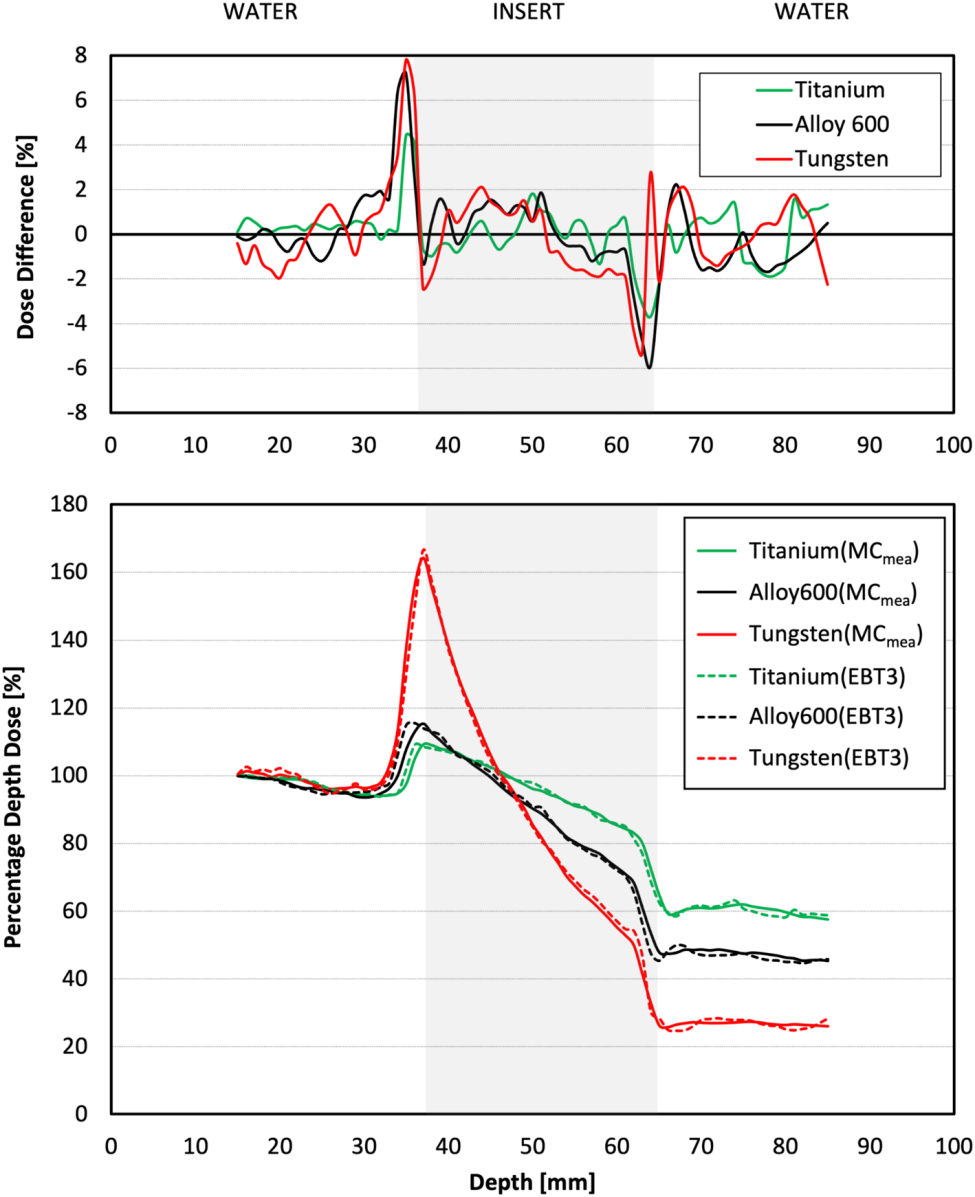
The comparison of dose differences and PDDs for measured (EBT3) and simulated (MC_mea_) PDDs for titanium, alloy 600, and tungsten inserts.

Measured PDDs agree well with those simulated for every insert (Fig. 3) with mean PDD values strongly correlated and not statistically different (*p* > 0.618, *R* > 0.998) (Table 1). Regional analysis show that the highest differences between measured and simulated doses were observed in the regions of insert edges. The maximum difference was 8% and was detected for the input edge of the tungsten insert (Fig. 3). The highest mean differences were seen on the input (3%) and output (− 3%) edges of alloy 600 insert (Table 1). In other regions, the maximum differences were within ± 2%, and the mean differences were lower than 1% for every insert.

**Table 1:** Measured (EBT3) and simulated (MC_mea_: Monte Carlo; measurement conditions) percentage depth doses, and mean dose difference (EBT3—MC_mea_) for selected regions.

	Titanium	Alloy 600	Tungsten
**Mean dose difference (and standard deviation) [%]**			
Water in front of the insert	0.3 (0.3)	0.2 (1.0)	− 0.1 (1.2)
Input edge of the insert (± 2 mm)	1.4 (2.6)	3.0 (3.7)	2.6 (4.6)
The insert	0.1 (0.7)	0.3 (1.0)	− 0.1 (1.4)
Output edge of the insert (± 2 mm)	− 2.1 (1.6)	− 3.0 (2.6)	− 1.6 (3.4)
Water behind the insert	0.0 (1.2)	− 0.6 (1.0)	0.1 (1.2)
**Comparison of whole PDDs (from 15 to 85 mm)**			
Similarity of distribution *(Kołmogorov–Smirnov test)*	*p* = 0.962	*p* = 0.758	*p* = 0.618
Coefficient of correlation *(Pearson corelation test)*	R = 0.998	R = 0.998	R = 0.999

### MC_mea_ versus MC_pla_ comparison

**Figure 4:**
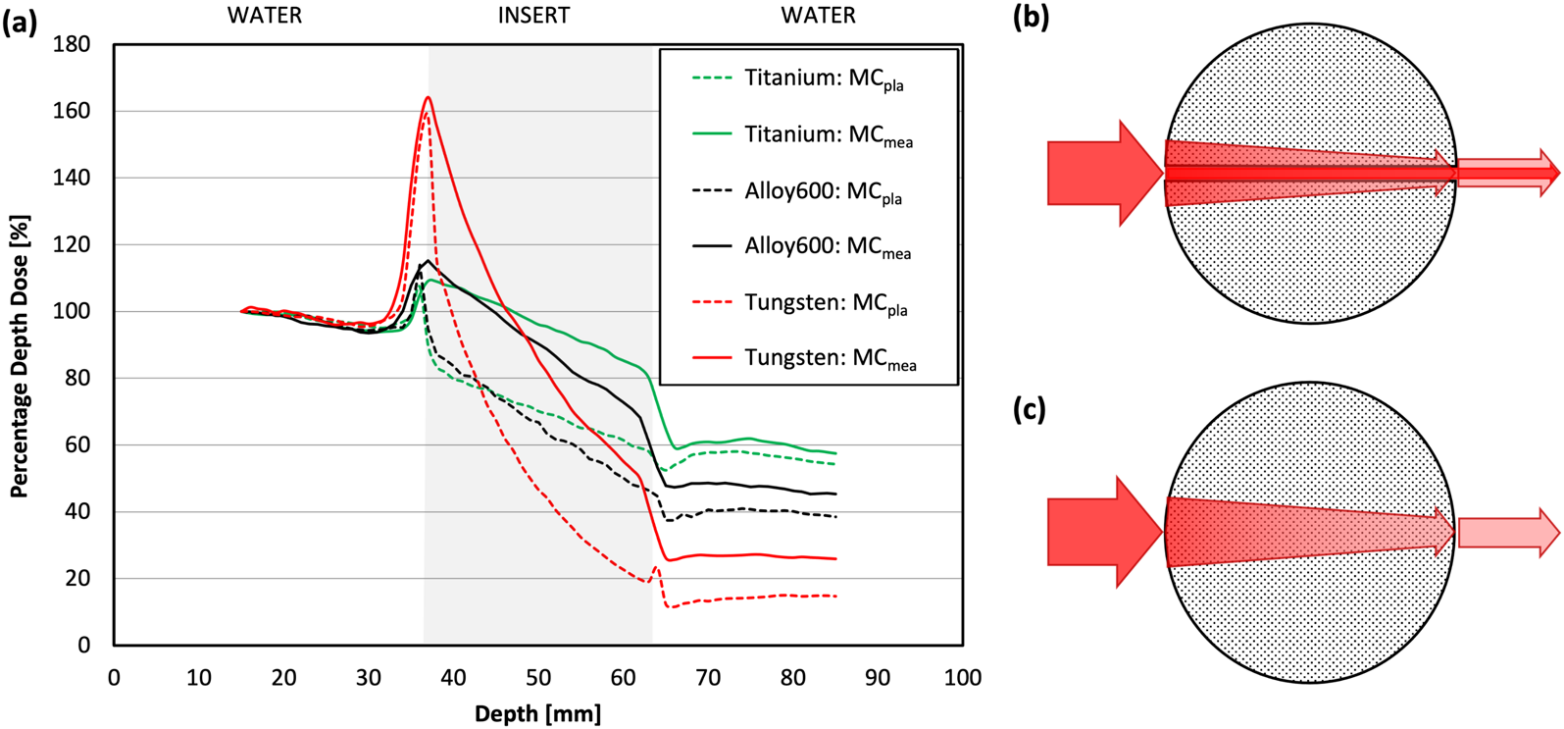
(**a**) The comparison of PDDs obtained from Monte Carlo simulations performed for measurement (MC_mea_) and TPS conditions (MC_pla_) for titanium, alloy 600, and tungsten inserts. Visualisation of the insert geometry, (**b**) two halves of the grey circle with a film gap between them (MC_mea_ conditions) and (**c**) solid grey circle without film gap (MC_pla_ conditions). The red arrows visible in (**b**) and (**c**) represent the radiation beam.

The PDDs simulated for measurement condition, MC_mea_, significantly differ from those simulated for TPS condition, MC_pla_ (*p* < 0.002) (Fig. 4a). The PDD values in the insert region for MC_pla_ (Fig. 4c) were on average lower by 23% (titanium), 26% (alloy 600), and 37% (tungsten) than corresponding MC_mea_ values (Fig. 4b). In the region behind the insert, PDDs from MC_pla_ were on average lower by 4% (titanium), 7% (alloy 600), and 12% (tungsten) than PDDs from MC_mea_.

### MC_pla_ versus TPS comparison

**Figure 5:**
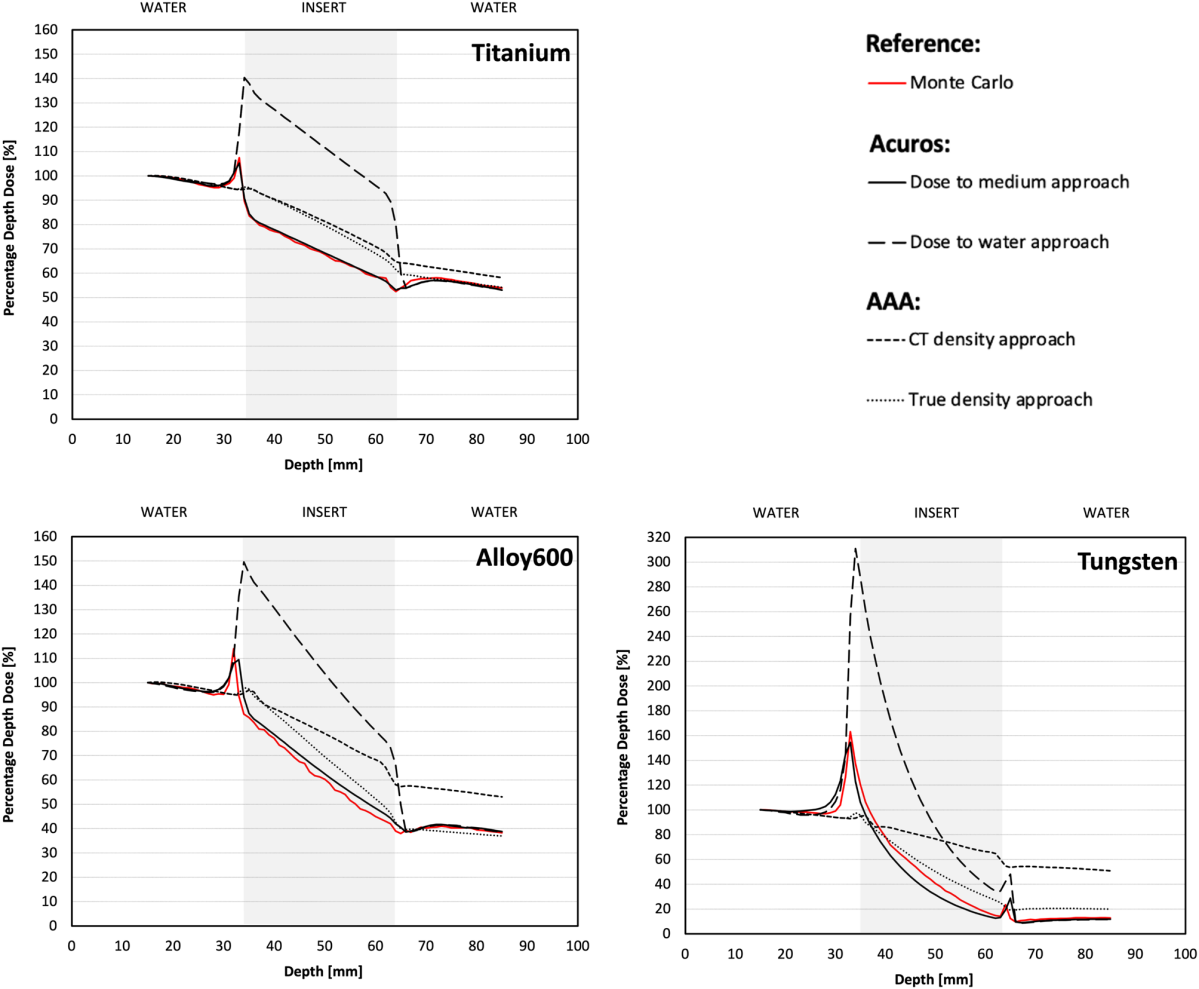
The comparison between PDDs obtained from Monte Carlo simulations performed for TPS condition (red line) and PDDs obtained from TPS calculations on standard CT reconstruction using different algorithms for titanium, alloy 600, and tungsten inserts.

The best match to the MC_pla_ PDDs was observed for the PDDs calculated by the Acuros XB algorithm based on the dose-to-medium approach (AXB_DM_) (Fig. 5). PDDs obtained from AXB_DM_ calculations were similar to MC_pla_ PDDs regardless of the CT reconstruction methods and inserts used (*p* > 0.078, *R* > 0.987) (Table 2). In contrast, PDDs obtained using other algorithms significantly differ from the MC_pla_ PDD (*p* < 0.011).

**Table 2:** The statistics of similarity between calculated (AXB_DM_: Acuros XB; dose-to-medium approach) and simulated (MC_pla_: Monte Carlo; TPS conditions) percentage depth doses, and mean dose difference (AXB_DM_ – MC_pla_) for selected regions of comparison.

Insert	Titanium					
Reconstruction	Standard CT		70 keV PMR		130 keV PMR	
Iterative metal artefact reduction	NO	YES	NO	YES	NO	YES
**Mean dose difference (and standard deviation) in compartments[%]**						
Water in front the insert	0.0 (0.4)	0.8 (0.6)	0.3 (0.6)	0.1 (0.7)	0.4 (1.1)	0.6 (0.8)
Input edge of the insert (± 2 mm)	0.6 (1.7)	1.3 (1.8)	1.4 (1.6)	1.1 (1.5)	2.2 (1.6)	1.8 (2.0)
The insert	0.5 (0.5)	1.7 (0.6)	0.8 (0.5)	1.4 (0.5)	1.8 (0.6)	2.4 (0.6)
Output edge of the insert (± 2 mm)	− 1.0 (1.2)	0.6 (1.3)	− 0.7 (1.3)	0.2 (1.5)	0.0 (1.7)	0.8 (1.4)
Water behind the insert	− 0.7 (0.3)	− 0.4 (0.3)	− 0.7 (0.3)	− 0.7 (0.3)	− 0.4 (0.2)	− 0.5 (0.2)
**Comparison of whole PDDs (from 15 to 85 mm)**						
*Similarity of distribution (Kołmogorov–Smirnov test)*	p = 0.618	p = 0.962	p = 0.880	p = 0.758	p = 0.882	p = 0.960
Coefficient of correlation *(Pearson corelation test)*	R = 0.999	R = 0.998	R = 0.999	R = 0.998	R = 0.997	R = 0.997

**Insert**	**Alloy 600**					
Reconstruction	Standard CT		70 keV PMR		130 keV PMR	
Iterative metal artefact reduction	NO	YES	NO	YES	NO	YES
**Mean dose difference (and standard deviation) in compartments[%]**						
Water in front the insert	0.4 (1.1)	0.5 (0.8)	1.3 (0.9)	1.1 (0.8)	0.9 (1.2)	0.9 (1.2)
Input edge of the insert (± 2 mm)	4.0 (7.6)	2.8 (8.5)	4.5 (8.4)	3.2 (8.9)	− 1.1 (4.7)	− 0.1 (3.6)
The insert	2.8 (0.8)	2.9 (0.8)	3.6 (0.7)	3.6 (0.7)	1.4 (0.9)	2.0 (0.9)
Output edge of the insert (± 2 mm)	1.0 (1.6)	2.2 (1.3)	1.7 (1.7)	2.5 (1.4)	0.7 (1.2)	1.8 (1.2)
Water behind the insert	0.6 (0.4)	0.4 (0.3)	1.3 (0.3)	0.9 (0.3)	− 0.3 (0.2)	0.1 (0.3)
**Comparison of whole PDDs (from 15 to 85 mm)**						
*Similarity of distribution (Kołmogorov–Smirnov test)*	p = 0.880	p = 0.618	p = 0.185	p = 0.187	p = 0.963	p = 0.758
Coefficient of correlation *(Pearson corelation test)*	R = 0.995	R = 0.995	R = 0.995	R = 0.995	R = 0.998	R = 0.998

**Insert**	**Tungsten**					
Reconstruction	Standard CT		70 keV PMR		130 keVPMR	
Iterative metal artefact reduction	NO	YES	NO	YES	NO	YES
**Mean dose difference (and standard deviation) in compartments[%]**						
Water in front the insert	2.4 (3.9)	1.1 (1.8)	1.8 (2.9)	0.7 (1.6)	2.0 (3.5)	1.2 (2.0)
Input edge of the insert (± 2 mm)	0.1 (17.2)	– 5.2 (11.5)	– 1.0 (14.0)	– 5 (11.4)	0.0 (17.3)	– 3.6 (11.7)
The insert	– 7.7 (3.1)	– 8.0 (3.2)	– 7.7 (3.0)	– 7.9 (3.2)	– 7.7 (3.0)	– 7.7 (3.0)
Output edge of the insert (± 2 mm)	1.9 (8.3)	1.3 (7.4)	1.3 (7.7)	0.8 (7.0)	1.3 (9.7)	1.1 (9.4)
Water behind the insert	– 1.3 (0.2)	– 1.3 (0.2)	– 1.3 (0.2)	– 1.4 (0.2)	– 1.8 (0.2)	– 1.9 (0.2)
**Comparison of whole PDDs (from 15 to 85 mm)**						
Similarity of distribution *(Kołmogorov–Smirnov test)*	*p* = 0.126	*p* = 0.104	*p* = 0.094	*p* = 0.084	*p* = 0.092	*p* = 0.078
Coefficient of correlation *(Pearson corelation test)*	R = 0.987	R = 0.991	R = 0.990	R = 0.992	R = 0.987	R = 0.991

The PDDs calculated by the Acuros XB algorithm based on the dose-to-water approach (AXB_DW_) was grossly overestimated in the insert region. The mean dose differences between the AXB_DW_ and the MC_pla_ PDDs in this region, depending on CT reconstruction mode, ranged from 43.6 to 45.0% for titanium, 43.0 to 45.6% for alloy 600, and 61.1 to 62.5% for tungsten. In the water regions (both in front and behind the inserts) the mean dose differences between AXB_DW_ and the MC_pla_ PDDs were lower than 2% for each CT reconstruction mode. The PDDs obtained from AAA calculations are more comparable to the MC_pla_ for the true density (AAA_TD_) than for the CT density (AAA_CT_) based approach (Fig. 5). In the region in front of the insert, both approaches of AAA dose calculations agree with MC_pla_ (differences < 2%). In the input/output edge of the insert due to lack of backscattered radiation, the differences between MC_pla_ simulation and doses calculated by both AAA methods were, respectively, up to 10% for titanium, 15% for alloy 600, and 50% for tungsten. In the region behind the insert better agreement to MC_pla_ was observed for AAA_TD_ than for AAA_CT_. The mean differences for AAA_TD_ versus MC_pla_ and AAA_CT_ versus MC_pla_ were, respectively, up to 1% and 3.5% for titanium; 2% and 10% for alloy 600; and 7.5% and 36% for tungsten. In the insert region, the mean differences between AAA_TD_ versus MC_pla_ were lower than AAA_CT_ versus MC_pla_ and were, respectively, up to 11% and 12% for titanium; 9% and 15% for alloy 600; and 6% and 25% for tungsten.

**Figure 6:**
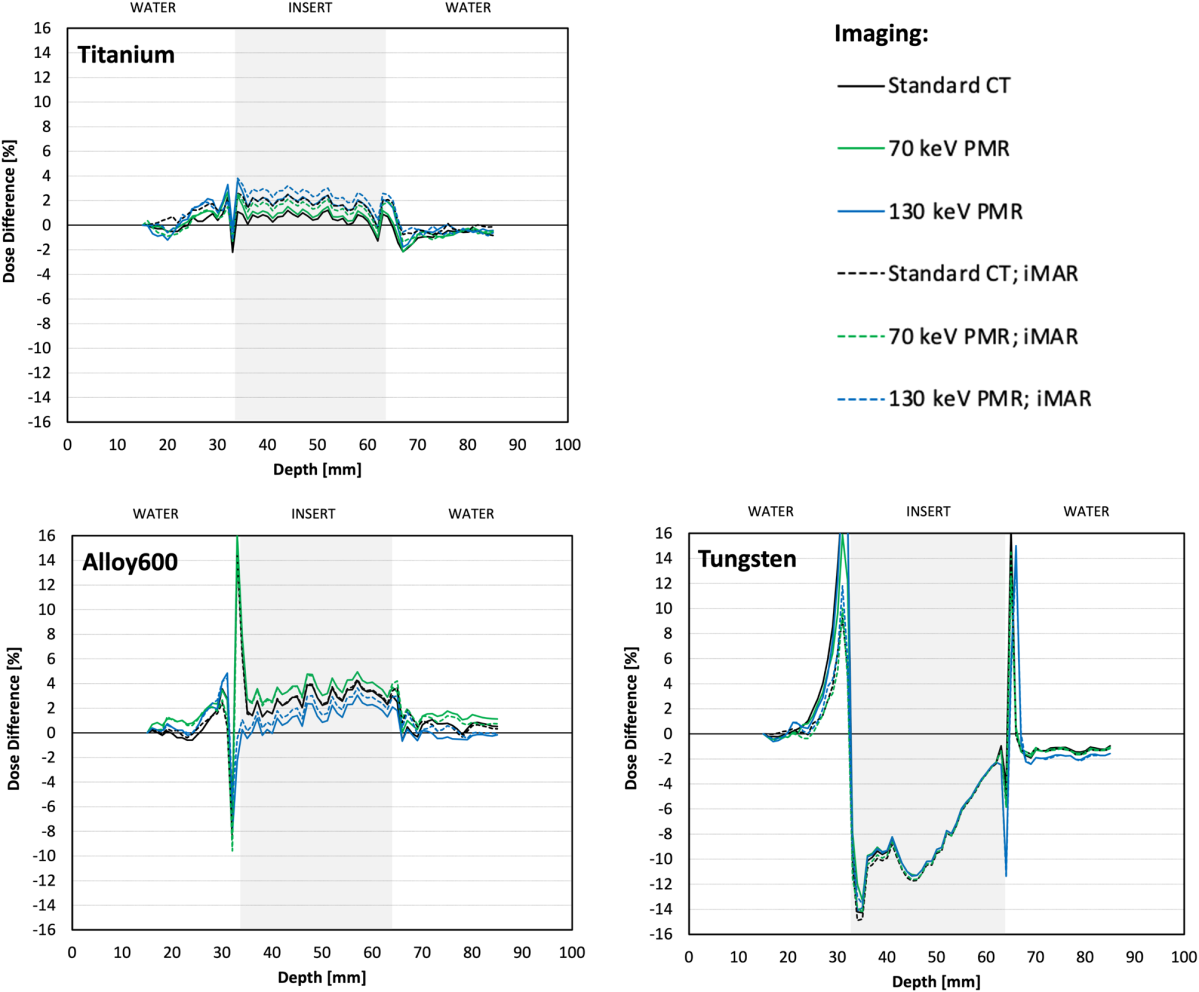
Differences between PDDs obtained from Monte Carlo simulations performed for TPS condition and PDDs obtained from TPS calculations using Acuros XB algorithm based on dose-to-medium approach on different CT reconstructions for titanium, alloy 600, and tungsten inserts, respectively.

Figure 6 shows the differences between MC_pla_ and AXB_DM_ PDDs obtained from calculations on different CT reconstructions for titanium, alloy 600, and tungsten inserts.

The highest differences between the MC_pla_ and the AXB_DM_ PDDs related to different CT reconstructions were observed at insert edges and they ranged from 4 to − 2% for titanium, 16% to − 9.5% for alloy 600, and 16% to − 15% for tungsten (Fig. 6). For the input edge regions standard deviations ranged from 1.5 to 2.0% for titanium, 3.6 to 8.9% for alloy 600, and 11.4 to 17.3% for tungsten (Table 2). In regions in-front and behind the insert, differences between the MC_pla_ and the AXB_DM_ PDDs were up to 2% (Fig. 6). The differences in the insert region depended on the density of the insert and were the lowest (up to 3%) for the titanium (4.5 g/cm^3^) and the highest (up to 12%) for the tungsten (19.4 g/cm^3^). Different CT reconstruction methods lead to differences between AXB_DM_ PDDs in all compartments ranging up to 2%. Nevertheless, there is no clear superiority of one method of reconstruction over the others (Fig. 6).

All sets of PDD curves calculated using different algorithms, CT reconstructions and inserts are presented in a supplementary material.

## Discussion

Several studies show that both algorithms (AAA and AXB) could accurately calculate the doses near the metal^25–27^. However, these studies focus specifically on spine SBRT (stereotactic body radiation therapy) treatment in the presence of titanium screws. Due to the relatively small dimensions of the screws and titanium density, the dose calculated in the tissues surrounding the screws is in line with measurements. Our results are in line with these observations, the doses calculated by AAA and AXB algorithms in the water near the titanium insert are close to MC simulations (Fig. 5). Nevertheless, there may be metal structures with a higher density than titanium in the patient body and geometries larger than screws used for spine stabilisation^13,16,28^. In general, to check accuracy between calculations and measurements, the ionising chambers or EBT films for in-axis dose measurements in front and behind the metal inserts and the perpendicularly oriented to the axis EBT films for profile measurements were used. Our study focused specifically on in-axis percentage depth dose measurements and calculations. In addition to previous studies, the EBT3 film placement in our phantom allows to simulate measurement conditions inside metal inserts. We recognise that the EBT3 films between the insert's halves resulted in measurements in a thin gap (< 0.3 mm) between parts of the insert filled by the film, rather than inside of the insert. Due to scanning modes used and the film thickness, the gap between the insert halves was not visible on the reconstructed CT images. Even with the smallest possible grid for dose calculation in TPS, the grid size was three times larger than the film thickness and the TPS calculations do not take into account the gap in the insert. Therefore, the measured doses by film were used only to compare to MC simulations for measurement conditions to prove the accuracy of MC simulations. The percentage depth doses obtained from MC simulation for TPS conditions (without the gap) were used as reference data for TPS calculations.

Analysing the accuracy of dose calculation through different algorithms shows the superiority of Acuros XB algorithm based on dose-to-medium approach over the other calculation methods in agreement with previous studies^16,29,30^. Another option of dose reporting mode in Acuros XB, dose-to-water, was also evaluated. Both approaches (dose-to-medium and dose-to-water) calculate the energy-dependent electron fluence based on the material properties of the interested media. Both approaches are based on the same steps of transport calculation^31^. The difference occurs in the post-processing step, during which the energy-dependent fluence resulting from transport calculation is multiplied by the different flux-to-dose response functions to obtain the absorbed dose value. Acuros XB uses a medium-based response function for dose-to-medium and a water-based response function for dose-to-water^32^. While our findings show comparable results of dose calculation around the insert for both Acuros XB approaches, the dose-to-water approach overestimates dose inside the inserts. The weak point of Acuros XB dose-to-medium approach is the rigid and non-modifiable list of high-density materials for which calculations can be made. In addition to previous studies, we performed calculations not only for titanium and stainless steel (i.e., alloy 600 in our study) but also for tungsten that is not listed in the algorithm's libraries. In order to perform calculations, we applied for the tungsten insert the closest density-similar material listed in the library, i.e., gold (19.3 g/cm^3^). The Monte Carlo simulations were performed for real tungsten density, i.e., 19.4 g/cm^3^ Therefore, the differences between the calculated and simulated doses in the tungsten insert are bigger than for the other inserts.

The worst results were observed for the AAA algorithm, which is in line with previous research^17^. The main limitation of this algorithm is the inability to model backscatter radiation deriving from high-density materials. This radiation generates a dose peak observed at the entrance to high-density material. Our work shows that only Monte Carlo and Acuros XB correctly model this phenomenon. However, it is worth noting that assigning an estimated one CT value for the metal (AAA_TD_) over the CT values calculated by the CT (AAA_CT_) scanner led to a better agreement with MC simulations.

This work also verified different strategies for metal artefact reduction. We assessed six different imaging methods: standard CT, monoenergetic CTs series reconstructed for 70 keV and 130 keV with and without the iMAR algorithm. Knowing how imaging affects the dose calculation is essential for choosing the proper metal artefact reduction method. Our study shows that the metal reduction strategies have no significant impact on the dose calculation results. Therefore, in our opinion, the selection of the most adequate CT reconstruction should be based on the preferences and experience of the person responsible for the delineation process. Dual-energy tomography and its monoenergetic reconstructions are an interesting metal artefact reduction method. One of the proposed methods is a combination obtained from dual-energy 70 keV monoenergetic scans with iMAR, which was reported^12^ to decrease CT number errors and increased image quality. Other work^33^ reported the lowest metal artefacts at 130 keV monoenergetic series without iMAR. It is important to highlight that the correct conversion from CT value to relative electron density or physical density is crucial.

A dedicated extended calibration curve should be determined and used for accurate dose calculation^34^. We found no significant impact of imaging mode on the dose calculation process with different strategies leading only to 2% differences and hence no disadvantages of using metal reduction strategies of choice.

This study, for the first time, compared detailed measurement around and inside the metal structure with a set of calculations by different methods using a dedicated phantom. There is a need in the future to translate and validate our phantom findings in clinical conditions.

## Conclusion

The selection of the algorithm for dose calculation was shown to have a significant impact on the accuracy of dose calculation near and inside metals. The Monte Carlo class algorithm should be used for the precise dose calculation as proved by our measurements with a phantom. We found that only the Acuros XB algorithm with a dose-to-medium approach provides comparable accuracy with Monte Carlo method for dose calculation in metal regions when using Eclipse treatment planning system. The limitation of this algorithm is the need to assign material from a predefined library for the high-density objects. Based on the tungsten insert results, it was found that the inability to indicate precise atomic composition leads to calculation errors. The inability of the AAA algorithm to model backscatter dose requires caution in its clinical use for patients with metal implants. However, we found that using an estimated CT value can improve AAA dose calculation behind the metal. No significant impact on the dose calculation was found for a range of metal reduction strategies, suggesting that the choice could be made following clinical operator preference.

## Supplementary Information

Supplementary Information.

## Data Availability

All data generated or analysed during this study are included in this published article and its supplementary materials except the selected data (i.e., phase space files) used during Monte Carlo simulations that are provided and licenced by Varian Medical Systems (Palo Alto, USA).
