# Supplementary material for: Accuracy of the doses computed by the Eclipse treatment planning system near and inside metal elements
Source: Sci Rep. 2022 Apr 8;12:5974. doi: 10.1038/s41598-022-10072-8 (PMC8993896; doi:10.1038/s41598-022-10072-8)
Supplement: Supplementary file 1 — Supplementary Information. [file 41598_2022_10072_MOESM1_ESM.docx]

**Supplementary material**

The PDD curves, calculated using each of the analysed algorithms for each CT reconstruction and for each insert.

**S1. Titanium**


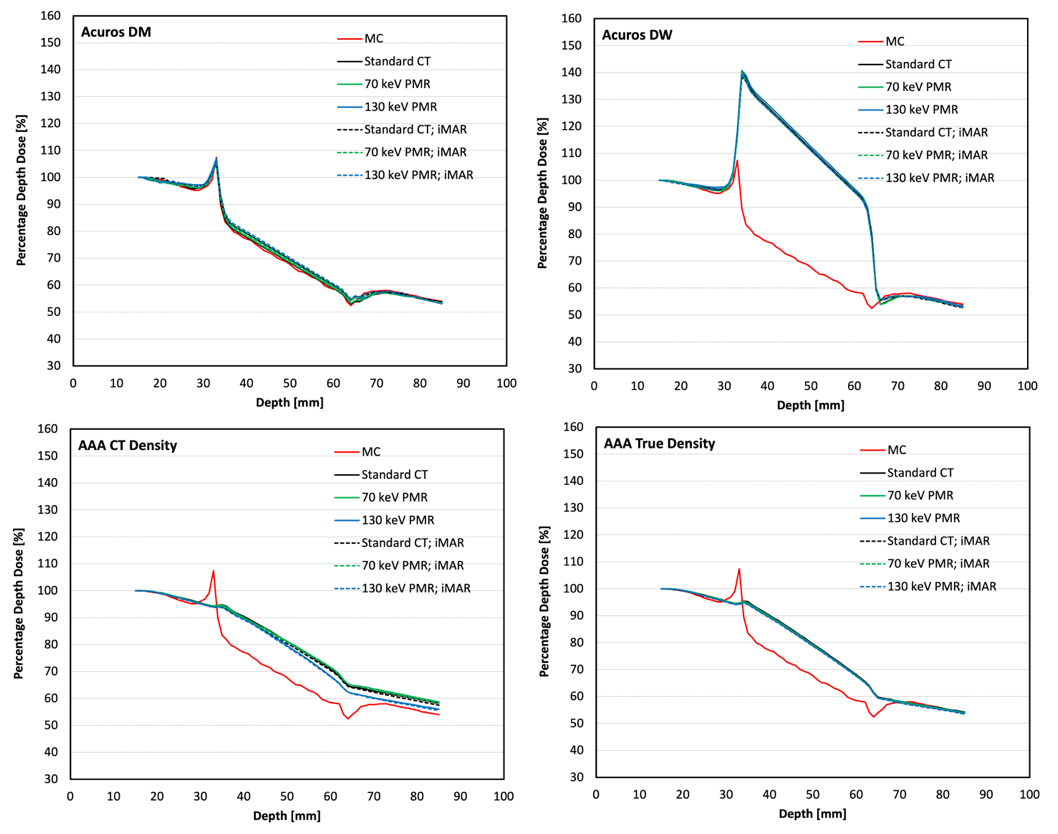


**S2. Alloy600**


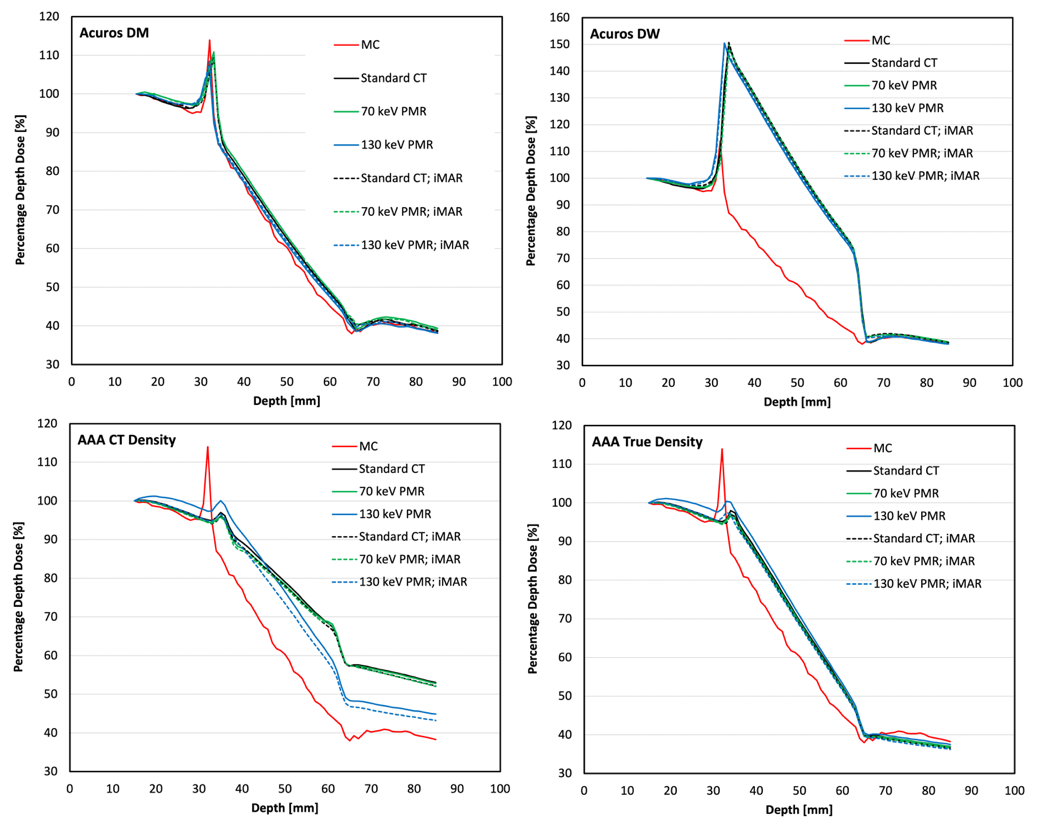


**S3. Tungsten**

**
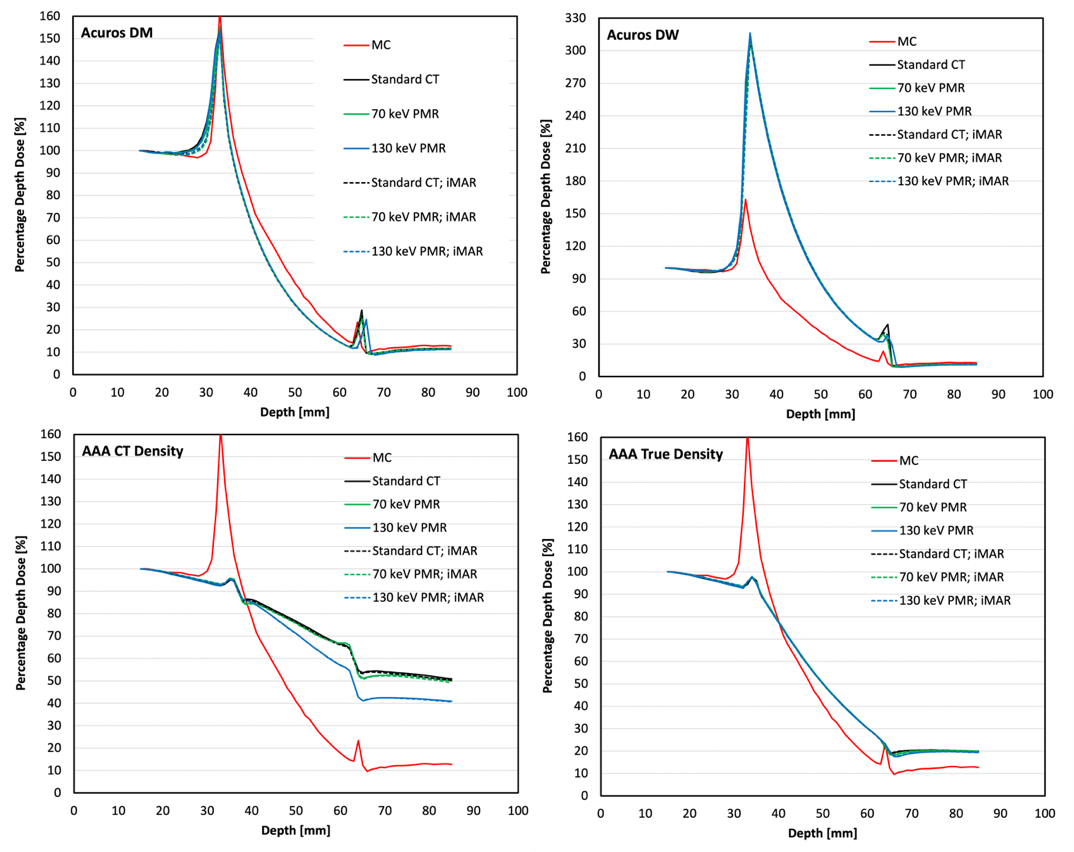
**
